# Long‐term results of palliative placement of very low‐axial force self‐expandable metallic stents for malignant colorectal obstruction

**DOI:** 10.1002/deo2.70126

**Published:** 2025-05-01

**Authors:** Rika Kyo, Takashi Sasaki, Shuntaro Yoshida, Hiroyuki Isayama, Tomonori Yamada, Toshiyuki Enomoto, Yorinobu Sumida, Toshio Kuwai, Masafumi Tomita, Takeaki Matsuzawa, Rintaro Moroi, Toshiyasu Shiratori, Yoshihisa Saida

**Affiliations:** ^1^ Department of Gastroenterology Saiseikai Yokohamashi‐Nanbu Hospital Kanagawa Japan; ^2^ Department of Hepato‐Biliary‐Pancreatic Medicine Cancer Institute Hospital of Japanese Foundation for Cancer Research Tokyo Japan; ^3^ Department of Endoscopy and Endoscopic Surgery Graduate School of Medicine The University of Tokyo Tokyo Japan; ^4^ Department of Gastroenterology Graduate School of Medicine Juntendo University Tokyo Japan; ^5^ Department of Gastroenterology Japanese Red Cross Aichi Medical Center Nagoya Daini Hospital Aichi Japan; ^6^ Department of Surgery Toho University Ohashi Medical Center Tokyo Japan; ^7^ Department of Gastroenterology National Hospital Organization Kyushu Medical Center and Clinical Research Center Fukuoka Japan; ^8^ Department of Gastroenterology National Hospital Organization Kure Medical Center and Chugoku Cancer Center Hiroshima Japan; ^9^ Department of Surgery Kobe Ekisaikai Hospital Hyogo Japan; ^10^ Department of Surgery Imusumiyoshi General Hospital Saitama Japan; ^11^ Division of Gastroenterology Tohoku University Hospital Miyagi Japan; ^12^ Department of Gastroenterology Kameda Medical Center Chiba Japan

**Keywords:** anti‐VEGF antibody, axial force, malignant colorectal obstruction, perforation, self‐expandable metallic stent

## Abstract

**Objectives:**

Stent placement is a standard option for palliative decompression in patients with malignant colorectal obstruction. Long‐term stent placement is associated with perforation, migration, and stent occlusion. Perforation is associated with life expectancy. Several studies have shown that stents with a high axial force (AF), which is defined as the force required to maintain the stent straight after it has bent, are associated with a higher perforation rate. Therefore, we evaluated the long‐term outcomes of using a very low AF stent (Niti‐S Enteral Colonic Uncovered stent D‐type) for palliative purposes.

**Methods:**

Eighty‐one consecutive patients with malignant colorectal obstruction in 33 medical institutions were evaluated. A stent with very low AF was placed using an endoscope system. We evaluated the adverse events (including perforation, migration, and stent occlusion), 1‐year survival rate, and cumulative patency rate. Univariate analysis was conducted using Fisher's exact test. The overall survival and cumulative patency rates were assessed using the Kaplan–Meier method.

**Results:**

The 1‐year cumulative survival rate was 37.8% after stent placement. The 3‐month, 6‐month, and 1‐year cumulative patency rates after stenting were 93.6%, 84.2%, and 75.8%, respectively. The major adverse events included stent migration (6.2%), stent occlusion (9.9%), and perforation (2.5%). Chemotherapy was administered in 26 cases (32.1%) after stenting, and bevacizumab was administered in five cases. However, no cases of perforation occurred following bevacizumab administration.

**Conclusions:**

Our results suggest that very low AF stents are safe and effective. Therefore, they may be a suitable option for applications, such as palliation. (UMIN 000011304)

## INTRODUCTION

In Japan, 10 years have passed since self‐expandable metallic stent placement was approved in January 2012, Colonic stents are now an option for palliative decompression in patients with malignant colorectal obstruction (MCRO). Long‐term stent placement is considered to be associated with intestinal perforation, stent migration, and stent occlusion. Intestinal perforation, in particular, is associated with a patient's life expectancy. However, the details of the long‐term results of palliative stent placement for MCRO are still limited.

The Niti‐S Enteral Colonic Uncovered Stent, D‐type (Taewoong Medical, Inc.) became available in Japan in July 2013. This stent is made of nitinol wire and woven into a hook‐and‐cross structure. Owing to its structure, the Niti‐S stent has an extremely low axial force (AF),^1^ defined as the force required to keep the stent straight after it has been bent.^2^ This stent is soft and conforms to the shape of the colon. Miyasako et al.^3^ reported that the perforation rate of the WallFlex colonic stent (high‐AF stent) was higher than that of the Niti‐S MD colonic metal stent (low‐AF stent). Some authors have reported that elevated AF owing to the use of the stent‐in‐stent technique can lead to intestinal perforation,^4^, ^5^ suggesting a correlation between AF and perforation rate.

We previously reported good short‐term results (within 7 days) after stent placement in a prospective study of 205 patients, which included bridge‐to‐surgery or palliative treatments using low‐AF stent (Niti‐S D‐type) insertion between October 2013 and May 2014.^6^ The technical and clinical success rates of the entire cohort were 97.5% and 96.0%, respectively. The major stent‐related adverse events included stent migration (1.0%), insufficient stent expansion (0.5%), and stent occlusion (0.5%). No intestinal perforation was observed. We hypothesized that the use of low‐AF stents would reduce the long‐term perforation rates. Therefore, we evaluated the long‐term outcomes (>8 days) of palliative low‐AF stent (Niti‐S D type) placement for MCRO based on the clinical success cohort from the previous multicenter study.

## METHODS

### Study population

**FIGURE 1:**
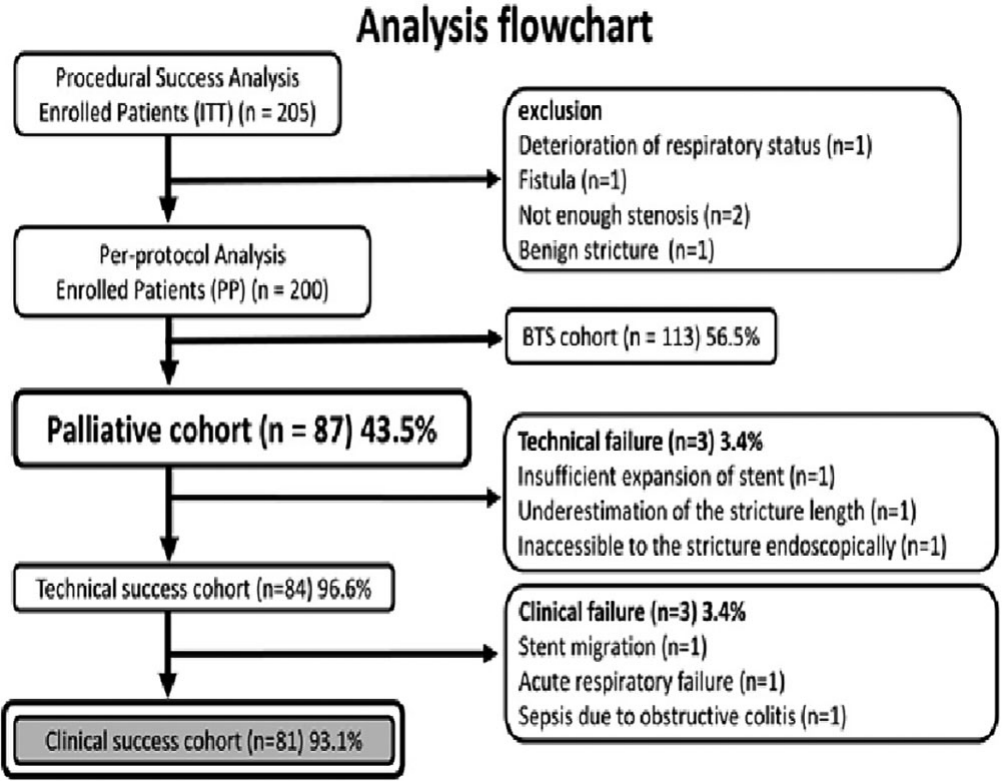
Flowchart of the Japanese multicenter study. BTS, bridge‐to‐surgery; ITT, intention‐to‐treat; MCRO, malignant colorectal obstruction; PP, per protocol.

This study evaluated the long‐term results of palliative stenting using the clinical success cohort from the previous Japanese multicenter study (Figure 1).^6^ and the study was approved by each institution's review board, informed consent was obtained from all patients. This study was performed per the guidelines of the Helsinki Declaration. The protocol was approved on August 19, 2013, at Toho University Ohashi Medical Center (protocol code: Ohashi 13–43), and the trial was registered with the University Hospital Medical Information Network Clinical Trial Registry (UMIN 000011304).

This multicenter study was a prospective, consecutive, observational, single‐arm clinical trial conducted at eight academic centers and 25 community hospitals. The study included both a bridge‐to‐surgery cohort and a palliative cohort. The short‐term results of the entire cohorts were previously reported. Patients who achieved short‐term clinical success were classified as the “Clinical success cohort.” Short‐term clinical success was defined as the complete disappearance of symptoms in a fasting state and relief from radiological findings within 24 h after stent placement. The study population in this analysis was the clinical success cohort from the palliative setting. Informed consent was obtained from all patients before the procedure, and registration was performed online through a website before or immediately after the procedure. Because colorectal stent placement is often urgent, online registration was allowed up to 1 hour after the procedure was completed. All unsuccessful procedures were also registered. Patient registration continued until study completion. The treatment intent (bridge‐to‐surgery or palliative) was determined based on the stage of malignancy, comorbidities, and the patients’ choices at enrollment. A computed tomography (CT) scan was performed before stent placement. Malignancy was diagnosed based on histopathological and endoscopic findings in all cases, and for an extrinsic origin, the primary tumor was identified using CT or other imaging modalities. Patients with colorectal obstructions or symptomatic strictures due to malignant neoplasms were eligible. In contrast, those with previous colonic stent placement, enteral ischemia, penetration, suspected or impending perforation, intra‐abdominal abscess caused by perforation, any contraindication to endoscopic treatment, and/or stent placement that deviated from the indications were excluded.

All patients received a Niti‐S Enteral Colonic Uncovered Stent, D‐type. The stent was 18 or 22 mm in diameter and 60, 80, 100, or 120 mm in length. This stent is made of nitinol wire woven into a hook‐and‐cross structure. By interlocking two strands of wire to create hooks, this design alleviates foreshortening and imbues the stent with conformability, allowing it to maintain its shape even in curved paths. The moving hook and its structure contribute to the stent's extremely low AF. The details of the stent placement procedure have been described in a previous report.^6^ Stent placement was performed or supervised by board‐certified endoscopists and surgeons from the Japan Gastroenterological Endoscopy Society.

### Definitions and data collection

Long‐term results were assessed from 8 days to 12 months after stent placement or until the last follow‐up or death, whichever occurred first.

The Colonic occlusive status was classified as complete or incomplete obstruction. Complete obstruction was confirmed based on the inability to pass flatus or water‐soluble contrast medium to the proximal part of the lesion or to visualize the proximal lumen endoscopically.^7^ The remaining cases were defined as incomplete obstruction.

The following data were prospectively evaluated: long‐term clinical success, stent‐related complications, and overall survival. Long‐term clinical success was defined as prolonged relief from symptoms until the end of follow‐up without the need for endoscopic or surgical reintervention or stent‐related mortality. Stent‐related complications requiring endoscopic or surgical re‐intervention included perforation, stent occlusion due to tumor ingrowth and overgrowth, and stent migration. Overall survival was calculated from the date of stent placement to the date of death or the last follow‐up.

### Statistical analysis

Statistical analyses were performed using EZR (Saitama Medical Center, Jichi Medical University, Saitama, Japan), a graphical user interface for R (The R Foundation for Statistical Computing, Vienna, Austria).^8^ The survival curves for overall survival and cumulative patency rate after stent placement were plotted using the Kaplan–Meier method. Risk factors for migration and stent occlusion were analyzed using univariate analysis with Fisher's exact test. The following nine factors were evaluated in the univariate analysis for migration and stent occlusion: age, sex, tumor site, obstruction state, tumor origin, length of stricture, length of stent, chemotherapy administered, and peritoneal dissemination. The level of statistical significance was set at p < 0.05.

## RESULTS

### Baseline Characteristics

From October 2013 to May 2014, 205 consecutive patients were enrolled. The observation period after stent placement was 1 year, and the average observation period was 211 days. A flowchart of the patient registration procedure is illustrated in Figure 1. Five patients were excluded. Among 87 patients in the palliative cohort, short‐term clinical success was achieved in 81 patients (93.1%).

Table 1 presents the baseline characteristics of the patients (*n* = 81), and Table 2 presents the tumor characteristics (*n* = 86) at study initiation for the clinical success cohort. Three patients had multiple strictures. Interventions before stent placement and procedure details are provided in Table 3. Intestinal decompression before stenting was performed in nine patients, five of whom had long intestinal tubes and four with transnasal decompression tubes. Preparation before stenting was performed in 42 patients, with 33 receiving enemas and six undergoing oral bowel cleansing. The median stricture length was 4 cm (interquartile range, 3–7 cm). The most commonly used stent length and diameter were 10 cm (31 patients; 34.1%) and 22 mm (90 patients; 98.9%), respectively.

**TABLE 1:** Patient characteristics (*n* = 81).

Sex (male/female)	39/42
Age (years)	79 (61–85)
ECOG performance status: 0/1/2/3/4	14/26/23/16/2
Symptoms of obstruction	
Deterioration of bowel habit	76 (93.8)
Bloating	64 (79.0)
Abdominal pain/cramping	62 (76.5)
Nausea or vomiting	36 (44.4)
CROSS before stent placement: 0/1/2/3/4	23/17/21/16/4
Previous treatment	
Chemotherapy/radiation therapy/none	23/2/57

Data are presented as *n*, *n* (%), or medians (interquartile ranges).

Abbreviations: CROSS, ColoRectal Obstruction Scoring System; ECOG, Eastern Cooperative Oncology Group.

**TABLE 2:** Tumor characteristics (*n* = 81).

Etiology of colorectal obstruction	
Primary colorectal cancer	58 (71.6)
Locally recurrent colorectal cancer	2 (2.5)
Other extrinsic origin	21 (25.9)
Gastric cancer	15 (18.5)
Pancreatic cancer	2 (2.5)
Bile duct cancer	1 (1.2)
Bladder cancer	1 (1.2)
Liposarcoma	1 (1.2)
Cancer of unknown primary	1 (1.2)
Status of obstruction: Complete/incomplete	64/17
Stricture: Single/multiple	78/3
Stenosis/tumor localization	*n* = 86
Rectum	12 (14.0)
Rectosigmoid junction	8 (9.3)
Sigmoid colon	16 (18.6)
Sigmoid‐descending junction	8 (9.3)
Descending colon	6 (7.0)
Splenic flexure	11 (12.8)
Transverse colon	10 (11.6)
Hepatic flexure	6 (7.0)
Ascending colon	4 (4.7)
Cecum	5 (5.8)
Metastatic site	
Liver	32 (39.5)
Lung	17 (21.0)
Peritoneal carcinomatosis	28 (34.6)
Others	18 (22.2)
Lymph node	11 (13.6)
Bone	2 (2.5)
Adrenal glands	1 (1.2)
Brain	1 (1.2)
Ovary	1 (1.2)
Pancreas	1 (1.2)
Pleural dissemination	1 (1.2)

Data are presented as *n*, *n* (%)

**TABLE 3:** Interventions before stent placement and procedure details.

Interventions	*n* = 81
Digestive tract decompression before stent placement	9 (11.1)
Long intestinal tube	5 (6.2)
Transanal‐decompression tube	4 (4.9)
Preparation	
Cleansing enema	33 (40.7)
Oral bowel cleansing	9 (11.1)
Stricture marking	
Intraluminal	31 (38.2)
Extraluminal	6 (7.4)
Number of strictures and stent placements	*n* = 91
Single stricture with one stent	74 (81.3)
Single stricture with two stents	2 (2.2)
Single stricture with three stents	2 (2.2)
Double stricture with two stents	2 (2.2)
Double stricture with three stents	1 (1.1)
Stent type	*n* = 91
6‐cm length, 22‐mm diameter	10 (11.0)
8‐cm length, 22‐mm diameter	26 (28.6)
10‐cm length, 22‐mm diameter	31 (34.1)
12‐cm length, 22‐mm diameter	23 (25.3)
6‐cm length, 18‐mm diameter	1 (1.1)
Stricture length (cm)	4 (3–7)

Data are presented as *n* (%) or medians (interquartile ranges).

### Efficacy and complications

Adverse events were observed in 20 patients (24.7%; Table 4). No stent‐related mortality after 8 days after stent placement. Moreover, intestinal perforation occurred in two patients. In one patient, bowel perforation developed just 8 days after stent placement, caused by the mechanical stress during the procedure to place an additional stent for stenosis at the oral side of the initially placed stent. In the other patient, perforation developed 38 days after stent placement during chemotherapy with panitumumab. Both patients underwent surgery and recovered from complications.

**TABLE 4:** Chronological changes in the occurrence of adverse events.

	Time of occurrence	
Adverse events	>8 days	>30 days
Stent migration (*n* = 5)	5	3
Gastrointestinal obstruction at the proximal site (*n* = 3)	3	3
Stent occlusion due to tumor ingrowth and/or overgrowth (*n* = 8)	8	7
Stent occlusion due to fecal impaction (*n* = 1)	1	1
Perforation (*n* = 2)	2	1
Minor bleeding (*n* = 1)	1	0

Migration occurred in five cases, with a median of 111 days (range, 19–212 days) to migration. All cases involved complete occlusion. Migration occurred during chemotherapy in four cases and radiation therapy in one case. In all cases, the stents were excreted with feces from the colon. After ≥30 days three migrations were observed—two during chemotherapy and one during radiation therapy.

Stent occlusion due to tumor ingrowth and/or overgrowth occurred in eight patients, with a median time to occlusion of 148.5 days (range, 21–361 days). Among the eight patients, five were treated with additional stent placement, two were managed surgically, and one was not treated surgically or endoscopically because of impending death. Stent occlusion due to fecal impaction occurred in one patient, and the feces were removed endoscopically. Stent occlusion occurred more frequently with increasing duration, with seven of eight cases occurring after 30 days.

Univariate analyses regarding stent migration and stent occlusion are presented in Tables 5 and 6, respectively. Stent migration was statistically significant in the chemotherapy group (odds ratio, 9.52; *p* = 0.03; 95% confidence interval [CI], 0.88–490.75), while stent occlusion was statistically significant in the colorectal cancer group (odds ratio, 0.20; *p* = 0.04; 95% CI, 0.03–1.15). We did not perform a univariate analysis for perforation due to the small number of perforation cases, which were insufficient for statistical analysis.

**TABLE 5:** Univariate analysis of the relationship between the clinicopathological factors and migration.

Clinicopathological factor	n	Odds ratio (95% CI)	*p*‐value
Age, years			
≥75	1	0.19 (<0.01–2.10)	0.17
<75	4	1	
Sex			
Male	3	1.66 (0.18–20.88)	0.67
Female	2	1	
Tumor site			
Left	5	Inf (0.55–Inf)	0.15
Right	0		
Obstruction state			
Complete	5	Inf (0.24–Inf)	0.58
Incomplete	0		
Tumor origin			
CRC	4	1.62 (0.15–83.82)	1
ECM	1	1	
Length of stricture			
≥4 cm	4	2.10 (0.19–108.43)	0.66
< 4 cm	1	1	
Length of stent			
≥10 cm	2	0.92 (0.03–3.86)	0.38
<10 cm	3	1	
Chemotherapy administered			
Yes	4	9.52 (0.88–490.75)	0.03
No	1	1	
Peritoneal dissemination			
Yes	2	1.25 (0.10–11.67)	1
No	3	1	

Abbreviations: CI, confidence interval; CRC, colorectal cancer; ECM, extracolonic malignancy; y, years

**TABLE 6:** Univariate analysis of the relationship between the clinicopathological factors and stent occlusion.

Clinicopathological factor	n	Odds ratio (95% CI)	*p*‐value
Age, years			
≥75	2	0.47 (0.07–2.64)	0.46
<75	6	1	
Sex			
Male	4	1.08 (0.19–6.30)	1
Female	4	1	
Tumor site			
Left	5	0.56 (0.10–3.26)	0.46
Right	3	1	
Obstruction state			
Complete	8	Inf (0.46–Inf)	0.19
Incomplete	0		
Tumor origin			
CRC	3	0.20 (0.03–1.15)	0.04
ECM	5	1	
Length of stricture			
≥4 cm	7	3.90 (0.46–184.90)	0.26
<4 cm	1	1	
Length of stent			
≥10 cm	6	2.07 (0.34–22.39)	0.47
<10 cm	2	1	
Chemotherapy administered			
Yes	4	1.30 (0.19–7.36)	0.71
No	4	1	
Peritoneal dissemination			
Yes	5	1.98 (0.34–11.64)	0.44
No	3	1	

Abbreviations: CI, confidence interval; CRC, colorectal cancer; ECM, extracolonic malignancy; y, years

**FIGURE 2:**
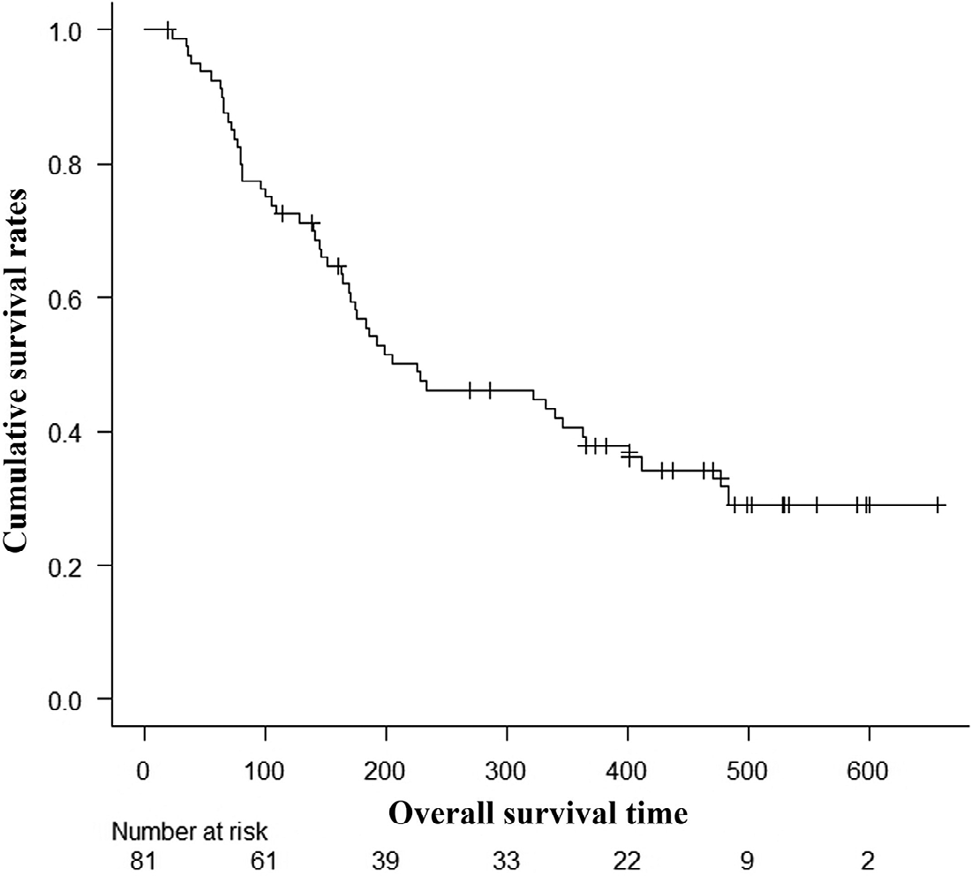
Cumulative survival rates after stent placement in patients with malignant colorectal obstruction (Kaplan–Meier method).

**FIGURE 3:**
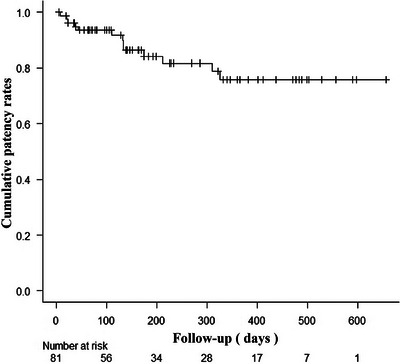
Cumulative patency rates 1 year after placement in malignant colorectal obstruction patients (Kaplan–Meier method).

The cumulative survival rate and the cumulative patency rate are shown in Figures 2 and 3, respectively. The 1‐year cumulative survival rate was 37.8% (95% CI, 0.270–0.486), and the median survival time was 226 days (95% CI, 170–363). The cumulative patency rate at 3 months, 6 months, and 1 year after stenting were 93.6% (95% CI, 0.852–0.973), 84.2% (95% CI, 0.722–0.913), and 75.8% (95% CI, 0.607–0.857) respectively.

### Anticancer treatment after stent placement

Chemotherapy was administered to 26 patients (32.5%), with 20 different regimens used after the stent placement (Table 7). Molecularly targeted drugs alone were administered to one patient with a single regimen, while cytotoxic chemotherapy regimens were administered to 19 patients using 13 different regimens. A combination of molecularly targeted treatment and cytotoxic chemotherapy was administered to seven patients with six different regimens. In five cases, adverse events related to chemotherapy were observed. Migration was observed in four patients, each of whom was treated with one of the following regimens: modified 5‐fluorouracil/leucovorin/oxaliplatin 6 + bevacizumab, paclitaxel (intraperitoneal injection), regorafenib, and carboplatin + S‐1 (tegafur/gimeracil/oteracil potassium), respectively. Moreover, perforation occurred in one patient treated with folinate/fluorouracil/irinotecan + panitumumab. The anti‐vascular endothelial growth factor antibody bevacizumab was administered to five patients using six different regimens. However, no perforation was observed following treatment with bevacizumab.

**TABLE 7:** Chemotherapy regimens after stent placement.

Regimens	*n*
mFOLFOX6	3
mFOLFOX6 + bevacizumab	2
FOLFIRI + bevacizumab	2
FOLFIRI + panitumumab	2
CapeOX	2
Capecitabine + bevacizumab	2
CPT‐11	2
CPT‐11 + cetuximab	1
Regorafenib	1
S‐1	3
SP	4
SOX	2
SOX + PTX (intraperitoneal injection)	2
S‐1 + bevacizumab	1
CBDCA + S‐1	1
Weekly PTX	1
nab‐PTX	1
PTX (intraperitoneal injection)	1
XELIRI	1
UFT/Uzel	1

Abbreviations: CapeOX, capecitabine/oxaliplatin; CBDCA, carboplatin; CPT‐11, irinotecan hydrochloride hydrate; FOLFIRI, folinate/fluorouracil/irinotecan; mFOLFOX6, modified 5‐ fluorouracil/leucovorin/oxaliplatin 6; nab‐PTX, nanoparticle albumin‐bound paclitaxel; PTX, paclitaxel; S‐1, tegafur/gimeracil/oteracil potassium; SOX, S‐1/oxaliplatin; SP, S‐1/cisplatin; XELIRI, capecitabine/irinotecan; UFT/Uzel, uracil/tegafur/folinate.

Radiation therapy was performed after stenting in five patients, and migration was observed in one patient who received radiation to the sigmoid colon.

## DISCUSSION

In the present study, we examined the long‐term clinical outcomes of palliative stent placement using very low AF stents in patients with MCRO. The perforation, migration, and stent occlusion rates were 2.5%, 6.2%, and 9.9%, respectively. These outcomes were better than those previously reported in palliative patients, where the perforation, migration, and stent occlusion rates ranged from 3.76% to 4.5%, 11% to 11.81%, and 7.34% to 12%, respectively.^9^, ^10^ Notably, intestinal perforation occurred in only two patients in the present study, and the occurrence rate was as low as 2.5%. The perforation rate after 30 days, limited to palliative cases, ranged from 4.1% to 7.6% in previous studies.^11^, ^12^, ^13^ In contrast, the perforation rate after 30 days was only 1.1% in this study.

In 2021, Sasaki et al. reported two methods for measuring AF. The first method involves bending the stent from a straight position to 60° and measuring the torque when bent.^1^ The second method involves bending the stent until the applied force remains < 0.05 mN/m and measuring the angle from the straight state. The Niti‐S D‐type stent has been classified into the group with the lowest AF among colonic stents available in Japan.^1^ As noted earlier, it has been suggested that stents with low AF have lower perforation rates.^1^, ^3^, ^4^, ^5^, ^6^, ^14^ Although no direct comparison was made between the very low AF stent and the high AF stent, the perforation rate was as high as 7% in the study by Ishibashi et al.^15^ which used the WallFlex, high AF stent. In contrast, the perforation rate in our study was 2.5%, which is lower than that reported by Ishibashi et al. Moreover, no cases of perforation were reported in palliative patients using the JENTLLY, very low AF stent.^16^ This may be one of the reasons for the lower perforation rate observed with stents having a low AF.

Intestinal perforation has been suggested to occur due to the use of anti‐angiogenic agents, which are not recommended following colonic stent placement according to the 2014 European Society of Gastrointestinal Endoscopy guidelines.^17^ However, in Japan we currently administer anti‐angiogenic agents depending on the condition of the tumor after obtaining informed consent. In the present study, six patients were treated with anti‐vascular endothelial growth factor antibodies, including bevacizumab in five patients and regorafenib in one patient. No cases of perforation occurred in these patients. Park et al.^18^ reported no significant difference in the perforation rate between patients treated with bevacizumab and those not treated with the drug. Furthermore, the 2020 European Society of Gastrointestinal Endoscopy guidelines^19^ have modified the protocol for the use of anti‐angiogenic agents in patients following colonic stenting. In the present study, intestinal perforation occurred in one patient treated with panitumumab, which is the anti‐epidermal growth factor receptor monoclonal antibody. Thus, the risk of intestinal perforation after treatment with any anti‐angiogenic agent should be considered in patients undergoing colonic stent placement. Moreover, migration occurred more frequently with statistical significance due to chemotherapy in this study. When chemotherapy is administered, attention should be given not only to the risk of perforation but also to the risk of migration.

A meta‐analysis by Ribeiro et al.^20^ noted that late stent dysfunction occurred in up to 19% of their patients. However, re‐canalization of stent dysfunction was typically performed via argon plasma coagulation, laser therapy, or additional stent insertion. The stent occlusion could be managed with endoscopic treatment. In this study, the cumulative patency rate at 1 year after stent placement was 75.8% and there was also a statistically significant difference in stent occlusion in the colorectal cancer group. Ingrowth and overgrowth obstructions of the tumor were treated surgically in two patients and endoscopically in five patients. In one of the two patients treated surgically, intestinal perforation occurred at another site, and surgery was performed. Thus, we considered that most cases of stent occlusion could be sufficiently re‐canalized via endoscopic treatment. A randomized controlled trial conducted by Fiori et al.^21^ revealed no significant difference in complications or mortality rates between colostomy and endoscopic stenting as palliative treatments for malignant rectosigmoidal obstruction. Ribeiro et al.^20^ also conducted a meta‐analysis of four randomized controlled trials. They observed no significant difference in the 30‐day mortality, mean survival period, or adverse events between emergency surgery and colonic stenting for MCRO in palliative cases. The results of the present study, especially regarding perforation, were less than those previously reported.^9^, ^10^, ^18^, ^20^, ^21^ Therefore, stent placement of very low AF as a palliative treatment for MCRO can be considered safe and effective.

The present study had some limitations. First, it was a single‐arm observational prospective study. Second, we did not directly compare stent placement with low AF to that with high AF. Third, we did not compare stent placement with surgical treatment, highlighting the need for an appropriate prospective randomized controlled trial.

In conclusion, our results suggest that very low AF stents are safe and effective. Therefore, low AF stents may be a suitable option for long‐term stenting, such as in palliative care.

## CONFLICT OF INTEREST STATEMENT

Takashi Sasaki received personal fees from Boston Scientific Japan, Co., Ltd., Century Medical, Inc., Cook Medical Japan, Kawasumi Laboratories, Inc., and Create Medic, Co., Ltd. Shuntaro Yoshida received personal fees from Boston Scientific Japan, Co., Ltd., Century Medical, Inc., and ZEON MEDICAL, Inc. Hiroyuki Isayama received personal fees from Boston Scientific Japan, Co., Ltd., Century Medical, Inc., TAEWOONG, Co., Ltd., FUJIFILM Healthcare, Co, Ltd., JAPAN LIFELINE, Co., Ltd., ZEON MEDICAL, Inc., Create Medic, Co., Ltd., Olympus, Co., Ltd., and Olympus Medical Science Sales Co., Ltd., received research grants from Boston Scientific Japan, Co., Ltd., Century Medical, Inc., FUJIFILM Healthcare, Co., Ltd., and ZEON MEDICAL, Inc., and received guidance fee of technique from Kawasumi Laboratories, Inc. Takeaki Matsuzawa received personal fees from Boston Scientific Japan, Co., Ltd. Yoshihisa Saida received personal fees from Boston Scientific Japan, Co., Ltd., and Century Medical, Inc., received grants from Boston Scientific Japan, Co., Ltd., Century Medical, Inc., Japan Lifeline, Co., Ltd., Kawasumi Laboratories, Inc., Create Medic, Co., Ltd., Zeon Medical, Co., Ltd., Olympus Medical Systems, Co., Ltd., and FUJIFILM Healthcare, Co., Ltd. and received consulting fees from Japan Lifeline, Co., Ltd., Olympus Medical Systems, Co., Ltd., and JIMPO, Co., Ltd. The other authors declare no conflict of interest.

## ETHICS STATEMENT

Approval of the research protocol by an Institutional Reviewer Board: The study was conducted according to the guidelines of the Declaration of Helsinki and was approved by the Ethics Committee of each institution. The protocol code is Ohashi 13–43, and the date and place of approval were August 19, 2013, in the Toho University Ohashi Medical Center.

## PATIENT CONSENT STATEMENT

Informed consent was obtained from all patients involved in this study.

## CLINICAL TRIAL REGISTRATION

The trial was registered with the University Hospital Medical Information Network Clinical Trial Registry (UMIN 000011304).
